# Application of statistical process control for monitoring bulk tank milk somatic cell count of smallholder dairy farms

**DOI:** 10.14202/vetworld.2020.2429-2435

**Published:** 2020-11-13

**Authors:** Veerasak Punyapornwithaya, Chalutwan Sansamur, Tawatchai Singhla, Paramintra Vinitchaikul

**Affiliations:** 1 Department of Food Animal Clinic, Faculty of Veterinary Medicine, Chiang Mai University, Chiang Mai, 50100, Thailand; 2 Veterinary Public Health Centre for Asia Pacific, Faculty of Veterinary Medicine, Chiang Mai University, Chiang Mai, 50100, Thailand; 3 Akkhraratchakumari Veterinary College, Walailak University, Nakorn Si Thammarat 80161, Thailand

**Keywords:** bulk milk somatic cell count, control chart, dairy farm, smallholder, statistical process control

## Abstract

**Background and Aim:**

Consistency in producing raw milk with less variation in bulk tank milk somatic cell count (BMSCC) is important for dairy farmers as their profit is highly affected by it in the long run. Statistical process control (SPC) is widely used for monitoring and detecting variations in an industrial process. Published reports on the application of the SPC method to smallholder farm data are very limited. Thus, the purpose of this study was to assess the capability of the SPC method for monitoring the variation of BMSCC levels in milk samples collected from smallholder dairy farms.

**Materials and Methods:**

Bulk tank milk samples (n=1302) from 31 farms were collected 3 times/month for 14 consecutive months. The samples were analyzed to determine the BMSCC levels. The SPC charts, including the individual chart (I-chart) and the moving range chart (MR-chart), were created to determine the BMSCC variations, out of control points, and process signals for each farm every month. The interpretation of the SPC charts was reported to dairy cooperative authorities and veterinarians.

**Results:**

Based on a set of BMSCC values as well as their variance from SPC charts, a series of BMSCC data could be classified into different scenarios, including farms with high BMSCC values but with low variations or farms with low BMSCC values and variations. Out of control points and signals or alarms corresponding to the SPC rules, such as trend and shift signals, were observed in some of the selected farms. The information from SPC charts was used by authorities and veterinarians to communicate with dairy farmers to monitor and control BMSCC for each farm.

**Conclusion:**

This study showed that the SPC method can be used to monitor the variation of BMSCC in milk sampled from smallholder farms. Moreover, information obtained from the SPC charts can serve as a guideline for dairy farmers, dairy cooperative boards, and veterinarians to manage somatic cell counts in bulk tanks from smallholder dairy farms.

## Introduction

Somatic cells are white blood cells presented in milk due to the defensive mechanisms of the mammary gland. The number of somatic cells or the somatic cell counts (SCCs) is the accepted international standard measurement of milk quality [1]. Bulk tank milk somatic cell count (BMSCC) is generally used as a parameter for the determination of milk quality. It depends on the number and duration of mastitis infection of the dairy herd [2]. The standard BMSCC that is considered to be good should not be >500,000 cells per mL (PMO, 2009) [3]. If the BMSCC level is higher than the standard threshold, the price paid for the milk is reduced [4]. Regarding the milk marketing system in Thailand [5,6], raw milk from the dairy farms is collected at milk collecting centers and subsequently transported to milk processing plants operated by private companies. The SCC level in the dairy cooperative bulk milk depends on the level of SCC in the milk obtained from its individual members. Therefore, the dairy cooperative boards have to manage the BMSCC level of the raw milk obtained from each dairy farm to meet the standard level with a low fluctuation using an appropriate tool.

The use of statistical process control (SPC) technique can be adopted by dairy cooperatives in Thailand to control the variations in the BMSCC. The SPC can be described as a process that uses statistical methods to monitor and control a process [7] to ensure an efficient operation for the given process. Important tools for the SPC include control charts, such as the individual value chart (I-chart) and the moving range chart (MR-chart) [8,9]. The SPC chart uses historical data to describe the performance of the process. The distribution of the output (e.g., BMSCC) in terms of mean and standard deviation (*σ*) can be used for the assessment of the output performance [10]. Several rules are applied for assessing out of control points and signals [9,11]. The most commonly used rules are the Shewhart and Western Electric rules [7,9,11]. The I-chart and MR-chart are commonly used in case of data that are continuous and not collected in subgroups. The I-chart displays the individual data and monitors mean and shift in the process while the variation is monitored by the MR-chart [11].

There is an increasing interest in SPC in livestock production [12]. The usefulness of SPC charts for obtaining dairy production data, such as milk yield and milk quality in some countries, is indicated by many reports [13,14], most of which demonstrate the implementation of SPC for large-scale dairy farms [14,15]. As the management systems of small-scale farms vary from large farms, it is useful to perform a study to examine the potential use of the SPC technique for small-scale farms.

Thus, this study aimed to assess the capability of the SPC method to determine variability, signal, and out of control observations for BMSCC obtained from smallholder dairy farms in Thailand.

## Materials and Methods

### Ethical approval

This type of study did not require ethical approval, as no animal use, the farm ID was slightly modified, and no questionnaire survey was performed.

### Dairy cooperative and farms

The study was conducted in a dairy cooperative located in Chiang Mai, North Thailand. Ninety farms were registered as members of the cooperative, most of which were small-scale dairy farms with an average number of 15-16 milking cows. The following similarities were identified among the farms: (1) Each farm used bucket type milking machine, (2) kept cows in tie-stall barn, (3) milked cows 2 times/day, and (4) fed cows with a commercial concentrate with seasonal roughage, such as corn and rice straw.

### Data

Data in association with the BMSCC were obtained from the dairy cooperative. These data were based on a regular system for testing the quality of the raw milk in bulk tanks transported from dairy farms. Bulk tank milk samples were collected 3 times/month (approximately every 7-10 days) for 14 months from January 2017 to February 2018. A sampling date was scheduled by the dairy cooperative board, which was blind to the farmers. Each of the milk samples was sent to the laboratory and subsequently analyzed on the same day after sampling. The level of BMSCC for each bulk tank milk sample was determined by an automated fluorescent microscopic somatic cell counter machine (Bentley Somacount 150, Bentley Instrument, USA).

### SPC charts

The I-chart and MR-chart statistical calculations are described in detail by Montgomery [11]. For I-chart, the average for individuals is calculated by the following formula:


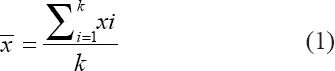


Where, *x_i_*=Value at point *i*

*k*=Number of individual data.

The formula for center line, lower control limit (LCL), and upper control limit (UCL) for I-chart is as follows:


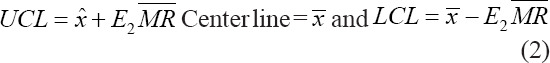


For MR-chart, the MR is the absolute value of the difference between consecutive measurements.


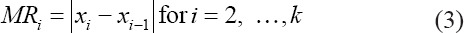


Mean *MR̄* is the average of all range value which is calculated by the following equation:


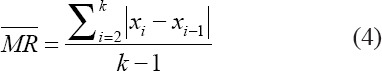


The UCL and LCL for MR-chart are calculated by the following formula


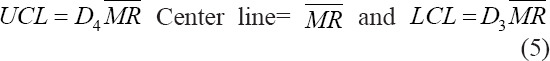


Where, D_3_, D_4,_ and E_2_ are the control chart constant.

The *t_i_* is the BMSCC value *t* from the sampling time *i*, where *i*=1,..,42. The BMSCC data from the first 4 months (*t*_1_,… *t*_12_) were used as prior data for constructing SPC charts. The SPC charts were created whenever the new BMSCC value was added without removing any prior data. Thus, the serial of SPC charts created for each farm can be written as a series of data (*t*_1_,… *t*_12_), (*t*_1_,… *t_13_*),…, (*t*_1_,… *t*_41_) and (*t*_1_,… *t*_42_).

### Interpretation of the SPC charts

The findings from the SPC charts were reported and interpreted each month to the dairy cooperative boards and veterinarians. The information obtained from the SPC charts and the conventional report was used to encourage dairy farmers to keep the level of BMSCC below the standard threshold and minimize variation.

### Statistical analysis

The distribution of the data on BMSCC for dairy farms was presented using boxplots. The SPC charts including the I-chart and the MR-chart for the data on BMSCC were created using *qichart* packages [16] from the R statistical software version 3.4.2 (R Foundation for Statistical Computing, Vienna, Austria)[17].

## Results

**Figure-1:**
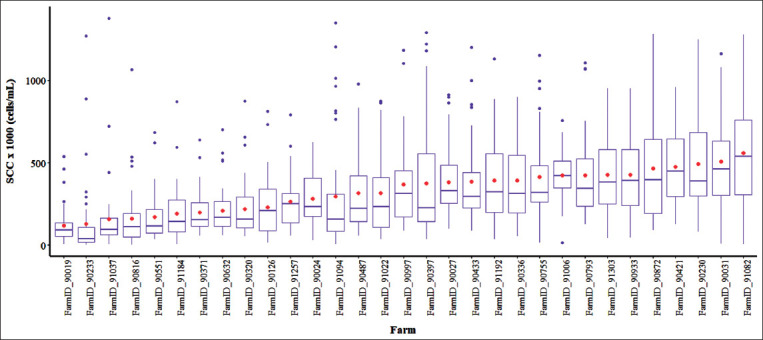
Distribution of bulk tank milk somatic cell count for each farm. A red dot inside a boxplot indicates mean value.

The distribution values of BMSCC for each farm are descriptively presented in Figure-1. It was clearly shown by this figure that most of the farms had a large variation in BMSCC, which was especially observed in case of farms with a high level of BMSCC. Moreover, several extreme values or values outside the boxplot interval could be observed. None of the farms had an average of BMSCC >500,000 cells/mL.

**Figure-2:**
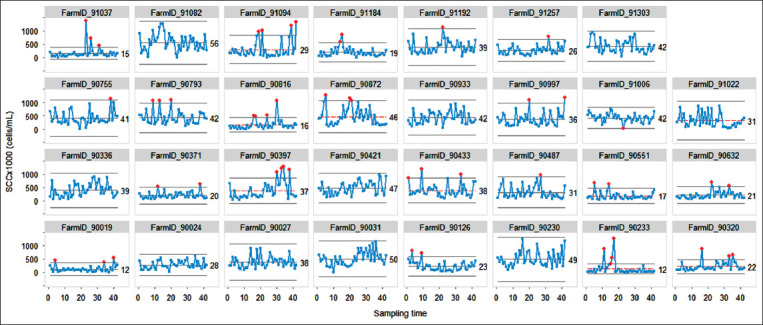
Individual charts for bulk tank milk somatic cell count (BMSCC; n=42 per farm) from 31 dairy farms. The X-axis refers to sampling times for bulk tank milk samples.

The I-charts for all the BMSCC data (*t*_1_,… *t*_42_) from each dairy farm are shown in Figure-2, which showed the variation of BMSCC for each farm over the entire study period with statistics including the center line, UCL, and LCL. If the interval between the UCL and the LCL was not large, the variation was low. Thus, the large majority of farms was identified to have a high variation in BMSCC (n=18; 58%). In contrast, there were 13 farms (42%) with a low variation in BMSCC. The examples of farms with a high variation of BMSCC were Farm ID 91082 and Farm ID90230, while the low variation of BMSCC was well defined for Farm ID 90019 and Farm ID 90233.

**Figure-3:**
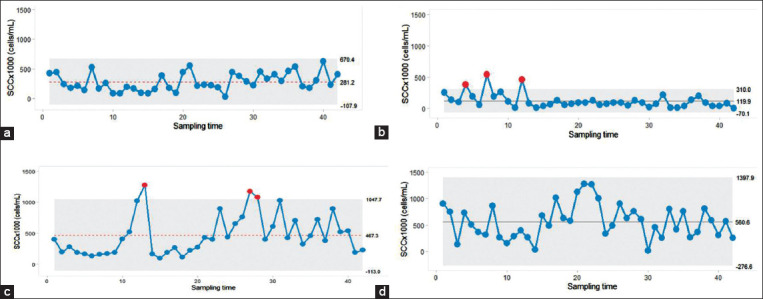
Individual charts demonstrate a dairy farm with the consistency in producing milk with low bulk tank milk somatic cell count (BMSCC) level and less in variation (a) whereas another farm had a high variation (b). An example of a farm with out of control point but low variation in BMSCC (c) and high variation in BMSCC (d) were shown. The horizontal line inside the gray shade indicates the center line of MR and the gray shade represents the upper and lower control limit. Red dots represent out of control points.

The ability of SPC charts to detect out of control points was demonstrated and shown in Figure-3.

The presence of out of control points more than 3 times during the entire study period was only confirmed for five farms. In addition, farms were categorized into four scenarios based on the level of variation and the existence of out of control points obtained from the SPC charts (Figure-3). The scenarios included farms with a high level of BMSCC variation and the lack of existence of out of control points (n=9), farms with a low level of BMSCC variation and the lack of existence of out of control points (n=1), farms with a high level of variation and the existence of out of control points (n=9), and farms with a low level of variation with the existence of out of control points (n=12).

**Figure-4:**
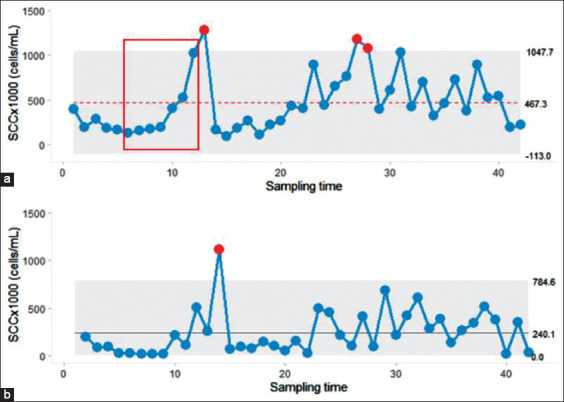
Based on the trend rule of statistical process control, more than 5 consecutive points of bulk tank milk somatic cell count shown an increasing trend before out of control point shows up on individual chart (a) but not for moving range chart (b). The horizontal line inside the gray shade indicates the center line of moving range and the gray shade represents the upper and lower control limit. Red dots represent out of control points.

**Figure-5:**
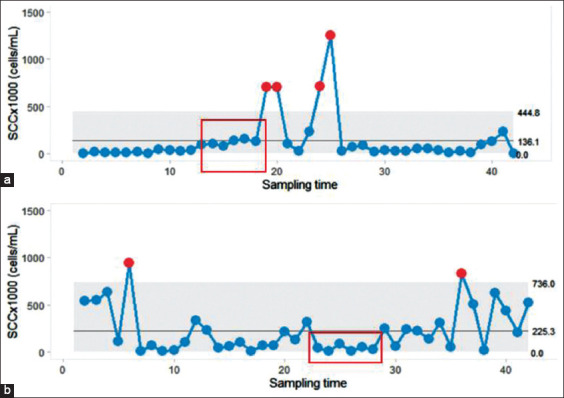
A series of bulk tank milk somatic cell count shows the signal corresponding to shift rule as more than 5 consecutive data points are located below the center line (dots in red box) in moving range chart (a). A false alarm was demonstrated (b) as no out of control point exists, although five consecutive data points (dots in red box) follow shift rule. The horizontal line inside the gray shade indicates the center line of moving range and the gray shade represents the upper and lower control limit. Red dots represent out of control points.

The SPC charts also provided signals or alarms, including signals corresponding to the trend and shift rule. An example of process shift is shown in Figure-4a when more than 5 consecutive BMSCC data were above or below the center line. Furthermore, the scenario in which the BMSCC data followed the trend rule was observed when more than 5 consecutive BMSCC data continued to increase (Figure-5a).

The dairy cooperative boards and veterinarians used the information obtained from the SPC charts and the conventional report to communicate with dairy farmers to encourage them to keep the level of BMSCC below the standard threshold and minimize the variation.

## Discussion

The generated SPC charts provide insights into the process variation overtime with statistics that can be used in the identification of periods when the process goes out of control. This study demonstrated the significance of the application of SPC charts in monitoring the variation of BMSCC values of milk obtained from smallholder dairy farms.

The SPC charts shown in Figure-2 have a benefit over the conventional chart (Figure-1), namely, that the variations in the BMSCC can be continuously observed as a time series pattern. Overall, some farmers sold bulk milk to the dairy cooperative with less variation in the BMSCC (Figure-2). An example of a farm with a good process control is shown in Figure-3a. This example farm produced milk with a low level of BMSCC and less variation as all of the bulk tank milk samples were identified to have BMSCC within the control threshold. A similar finding was observed on another farm, despite that the presence of out of control points was demonstrated (Figure-3b). In contrast, there is a scenario that some farmers continue to produce raw milk with high BMSCC levels and variation (Figure-3c and 3d). This condition is considered to be worse as the farmers regularly sell milk with high BMSCC levels and variation to the dairy cooperative, although the out of control points were shown (Figure-3c) or not shown (Figure-3d). Since the values of the BMSCC were depicted as a time series approach by the SPC charts, farmers can use the information from the SPC charts to set a strategy for achieving the desired level of BMSCC levels with a low variability.

It is suggested by the out of control BMSCC point that a problem impacts the process (e.g., having more cows with high SCC levels). With this insight, it is appropriate to identify the problem and address it. The milking management practice should be assessed by each farmer to identify milking cows with high SCC that causes an elevation of SCC in the bulk tank milk. The main advantage of the SPC charts is their use as an early warning indicator. Therefore, several rules for the identification of warning signals were reported to be applied to SPC charts [7,9,11]. For example, before the out of control point occurs, the presentation of more than 5 consecutive BMSCC values that continuously increased in line as well as matched a trend rule description [9] was defined and shown in Figure-4a. However, the out of control point may exist without prior alarm or signal. In contrarily, a false alarm occurs when the SPC charts produce a signal but none of the control points exist (Figure-5b). This situation might not be harmful to the dairy farmers as it will draw attention for the operator to examine the process. For some situations, the signal may be found in the I-chart (Figure-4a) but not for the MR-chart (Figure-4b) or vice versa. Thus, both the I-chart and the MR-chart should be constructed with the same data set to increase the sensitivity of the SPC.

Unlike several manufacturing products that require much effort in evaluating the process, dairy farmers can determine the cause of BMSCC values that are over the UCL with less effort. As the BMSCC is increased by pooling milk into the bulk tank from at least one cow with a high SCC due to an intramammary infection [18,19], farmers can tackle this problem by checking the individual SCC level for each milking cow. Milk removal from infected cows will solve this problem. On the other hand, BMSCC values lower than the LCL is the improvement of the milk quality as the level of the SCC is in a favorable direction. Thus, regarding the enhancement of the management system, dairy farmers may use SPC charts to monitor the level of BMSCC to the target level and subsequently sustain that level with less variability to archive better prices.

In this paper, the SPC chart is proposed as an additional tool for BMSCC monitoring in dairy cooperatives based on our findings. The SPC can be considered as an appropriate tool due to that it presents graphical data that can be easily interpreted by dairy farmers, dairy cooperative boards, and veterinarians. In addition, the application of this method is not expensive as the purchase of expensive commercial software is not required as the R software and its packages are free and can be used to build the SPC charts. Minimal resources are required, including the training of individuals (e.g., cooperative manager, veterinarian, and scientist) to be able to use the methods described in this study, including the interpretation of the derived results and communicating the information to stakeholders who can also use the software, interpret the results, as well as a set up a reporting system that delivers the results to the dairy farmers. However, it is important to note that SPC is a method for monitoring process variation and it is not intended to detect out of control causes [20,21]. The absence of an accurate alarm [22] indicator may cause the loss of trust in this system among dairy farmers. Besides, the parameters of the control limit should be evaluated and modified to achieve the output target.

## Conclusion

This study can be considered the first in Thailand; it demonstrated the capability and usefulness of the SPC charts in association with monitoring the level of SCC in bulk tank milk from smallholder farms. Our findings revealed that this method can detect the variation of BMSCC, out of control points, signals, and alarms. The implementation of SPC to the dairy cooperative is not very costly as it only requires basics resources, including computer hardware, free software for the creation of the SPC charts, and some training for the dairy cooperative staff or veterinarians. The approach used in this study can be applied to other dairy cooperatives or dairy organizations in other countries with similar systems.

## Authors’ Contributions

VP designed and administered the entire research work. VP and CS analyzed the data. TS and PV performed field works. VP wrote the manuscript. CS, TS, and PV reviewed and revised the text. All authors read and approved the final manuscript.

## References

[1] Rainard P, Foucras G, Boichard D, Rupp R (2018). Invited review: Low milk somatic cell count and susceptibility to mastitis. J. Dairy Sci.

[2] Reneau J.K, Lukas J (2006). Using statistical process control methods to improve herd performance. Vet. Clin. North Am. Food Anim. Pract.

[3] PMO (2009). Grade “A” Pasteurized Milk Ordinance. Department of Health and Human Services, Public Health Service. Food and Drug Administration USA.

[4] Troendle J.A, Tauer L.W, Grohn Y.T (2017). Optimally achieving milk bulk tank somatic cell count thresholds. J. Dairy Sci.

[5] Jitmun T, Kuwornu J.K.M, Datta A, Anal A.K (2019). Farmers'perceptions of milk-collecting centres in Thailand's dairy industry. Dev. Pract.

[6] Yeamkong S, Koonawootrittriron S, Elzo M.A, Suwanasopee T (2010). Milk quantity, quality and revenue in dairy farms supported by a private organization in central Thailand. Livest. Res. Rural Dev.

[7] Anhoj J, Wentzel-Larsen T (2018). Sense and sensibility: On the diagnostic value of control chart rules for detection of shifts in time series data. BMC Med. Res. Methodol.

[8] de Vries A, Reneau J.K (2011). Application of statistical process control charts to monitor changes in animal production systems. J. Anim. Sci.

[9] Koutras M.V, Bersimis S, Maravelakis P.E (2007). Statistical process control using shewhart control charts with supplementary runs rules. Methodol. Comput. Appl. Probab.

[10] Woodall W.H, Montgomery D.C (1999). Research issues and ideas in statistical process control. J. Qual. Technol.

[11] Montgomery D.C (2012). Introduction to Statistical Quality Control.

[12] Martens K, Decuypere E, de Baerdemaeker E, de Ketelaere B (2011). Statistical control charts as a support tool for the management of livestock production. J. Agric. Sci.

[13] Thysen I (1993). Monitoring bulk tank somatic cell counts by a multi-process Kalman filter. Acta Agric. Scand. Section - A Anim. Sci.

[14] Lukas J.M, Hawkins D.M, Kinsel M.L, Reneau J.K (2005). Bulk tank somatic cell counts analyzed by statistical process control tools to identify and monitor subclinical mastitis incidence. J. Dairy Sci.

[15] Niza-Ribeiro J, Noordhuize J.P, Menezes J.C (2004). Capability index: A statistical process control tool to aid in udder health control in dairy herds. J. Dairy Sci.

[16] Anhoej J (2017). Qicharts: Quality Improvement Charts. R Package Version 0.5.5.

[17] R Core Team. (2017) R: A Language and Environment for Statistical Computing. R Foundation for Statistical Computing, Vienna, Austria https://www.r-project.org.

[18] Wang B, Wang Y.J, Qin Y, Vallverdu R.G, Garcia J.M, Sun W, Li S, Cao Z (2016). Prevalence of bovine mastitis pathogens in bulk tank milk in China. PLoS One.

[19] Olatoye O, Amosun A, Ogbu U, Okunlade Y (2018). Bulk tank somatic cell count and associated microbial quality of milk from selected dairy cattle herds in Oyo State, Nigeria. Ital. J. Food Saf.

[20] Lim S.A.H, Antony J, Arshed N, Albliwi S (2017). A systematic review of statistical process control implementation in the food manufacturing industry. Total Qual. Manage. Bus. Excell.

[21] Suman G, Prajapati D (2018). Control chart applications in healthcare: A literature review. Int. J. Metrol. Qual. Eng.

[22] Hubig L, Lack N, Mansmann U (2020). Statistical process monitoring to improve quality assurance of inpatient care. BMC Health Serv. Res.

